# Enhanced syndromic surveillance for mass gatherings in the Pacific: a case study of the 11th Festival of Pacific Arts in Solomon Islands, 2012

**DOI:** 10.5365/WPSAR.2016.7.1.004

**Published:** 2016-09-27

**Authors:** Damian Hoy, Salanieta T Saketa, Roy Roger Maraka, Alison Sio, Ian Wanyeki, Pascal Frison, Divi Ogaoga, Dennie Iniakawala, Cynthia Joshua, Sala Duituturaga, Christelle Lepers, Adam Roth, Paul White, Yvan Souares

**Affiliations:** a Research, Evidence and Information Programme, Public Health Division, the Pacific Community.; b National Referral Hospital, Solomon Islands.; c Ministry of Health and Medical Services (MHMS), Solomon Islands.; d Agence Sanitaire et Sociale de la Nouvelle.

## Abstract

Mass gatherings pose public health challenges to host countries, as they can cause or exacerbate disease outbreaks within the host location or elsewhere. In July 2012, the 11th Festival of Pacific Arts (FOPA), a mass gathering event involving 22 Pacific island states and territories, was hosted by Solomon Islands. An enhanced syndromic surveillance (ESS) system was implemented for the event. Throughout the capital city, Honiara, 15 sentinel sites were established and successfully took part in the ESS system, which commenced one week before the FOPA (25 June) and concluded eight days after the event (22 July). The ESS involved expanding on the existing syndromic surveillance parameters: from one to 15 sentinel sites, from four to eight syndromes, from aggregated to case-based reporting and from weekly to daily reporting. A web-based system was developed to enable data entry, data storage and data analysis. Towards the end of the ESS period, a focus group discussion and series of key informant interviews were conducted. The ESS was considered a success and played an important role in the early detection of possible outbreaks. For the period of the ESS, 1668 patients with syndrome presentations were received across the 15 sentinel sites. There were no major events of public health significance. Several lessons were learnt that are relevant to ESS in mass gathering scenarios, including the importance of having adequate lead in time for engagement and preparation to ensure appropriate policy and institutional frameworks are put in place.

## Introduction

Mass gatherings are congregations of large numbers of people in a specific location or locations for a defined period of time – examples are major sporting events or festivals. (*1*) Planned mass gatherings are common occurrences in Pacific Island countries and territories. One of the risk areas of these gatherings is the potential for communicable diseases to spread efficiently and rapidly, causing outbreaks or amplifying existing outbreaks in the host country. (*2*)

Solomon Islands hosted the 11th Festival of Pacific Arts (FOPA) from 1 to 14 July 2012 in Honiara, the country’s capital. It was the largest event ever hosted by Solomon Islands, involving more than 3000 artists and performers from around the Pacific. Most visitors arrived the week before the event and departed up to one week after the festival.

Syndromic surveillance (SS) is used for the early detection of and response to potential public health threats. (*3*, *4*) Case definitions in SS are based on clinical signs and symptoms rather than laboratory confirmation for the early detection of outbreaks while preserving limited resources. (*4*) Solomon Islands’ SS system, established in 2011, monitors four syndromes at the National Referral Hospital (NRH).

Enhanced syndromic surveillance (ESS) is increasingly being used for mass gatherings, although it generally has been limited to high-income countries. (*5*) ESS usually involves expanding the number of sentinel sites and syndromes tracked as well as moving from aggregated to case-based reporting and from weekly to daily reporting. In low- and middle-income countries, ESS for mass gatherings has the potential to strengthen existing SS systems in a sustainable manner. (*3*, *4*)

In 2012 the Solomon Islands Ministry of Health and Medical Services (MHMS) requested the Pacific Community (SPC) to provide technical assistance for ESS at the FOPA. The objectives were:

1. to provide a simple surveillance system for detecting and responding to disease outbreaks in a timely and effective manner,
2. to sustain the surveillance system improvements beyond the mass gathering.

This paper reports on that experience.

## Methods

The approach that SPC takes for ESS systems for mass gatherings has three stages: (1) preparation, (2) operation and (3) sustainability. These stages, as they were implemented for the FOPA, are described below.

### Stage 1 – preparation

#### Formalize agreement with MHMS

The Solomon Islands MHMS and SPC agreed that SPC would provide ESS during and around the FOPA, starting two months before the event.

#### System and disease risk assessment

The existing SS system was assessed two months before the event to identify its strengths and weaknesses for a mass gathering and areas of enhancement needed for ESS. The assessment included a literature review of the disease patterns within the country, an assessment of disease databases, a reflective self-assessment with the public health and laboratory surveillance teams, interviews with key informants and focus group discussions with key stakeholders. A risk assessment was conducted that included assessing: the size, duration and characteristics of the event; priority communicable diseases of concern; medical resources and surge capacity; and the political will of decision-makers.

### Stage 2 – operation

The plan for ESS was developed in May 2012. The ESS system commenced one week before the FOPA (June 25) and concluded eight days after the event (July 22). The system included:

- Data sources: Expanded from one to 15 sentinel sites in Honiara comprising the NRH, nine public clinics, two private clinics and three temporary clinics set up primarily for the FOPA.
- Syndromes: Existing syndromes were diarrhoea, acute fever and rash (AFR), prolonged fever (PF) and influenza-like illness (ILI). To increase the likelihood of capturing outbreak-prone diseases that are common in the region, acute fever and neurological symptoms (AFN), fever and jaundice (F&J) and heat-related illness (HRI) were added; watery diarrhoea (WD) and non-watery diarrhoea (NWD) were reported separately in the new list. The new list of eight syndromes is shown in **Table 1**. Although HRI is not outbreak prone, it was included due to the risk of it occurring.

**Table 1:** The eight syndromes endorsed for surveillance, 11th Festival of Pacific Arts, Solomon Islands, 2012

Syndrome	Case definition	Important diseases to consider	Threshold
Influenza-like illness (ILI)	Sudden onset of fever* plus cough and/or sore throat	Influenza; other viral or bacterial respiratory infections	No specific threshold set**
Prolonged fever (PF)	Any fever* lasting three or more days	Typhoid fever; dengue; leptospirosis; malaria; other communicable diseases	No specific threshold set**
Non-watery diarrhoea (NWD)	Three or more loose stools in 24 hours	Viral and bacterial gastroenteritis, including food poisoning, ciguatera fish poisoning	No specific threshold set**
Acute fever and rash (AFR)	Sudden onset of fever* plus acute non-blistering rash	Measles; dengue; rubella; meningitis; leptospirosis	No specific threshold set**
Watery diarrhoea (WD)	Three or more watery, loose stools in 24 hours	Cholera	1 case
Acute fever and neurological symptoms (AFN)	Sudden onset of fever* with neurological symptoms; altered mental state; confusion; delirium; disorientation; seizure	Meningococcal meningitis; viral meningitis; other viral encephalitis (e.g. West Nile virus)	1 case
Fever and jaundice (F&J)	Any fever* plus jaundice	Hepatitis A infection	1 case
Heat-related illness (HRI)	Dehydration due to heat; heavy sweating; paleness; muscle cramps; dizziness; headache; nausea or vomiting; fainting; extremely high body temperature (> 40 °C); rapid, strong pulse	Heat cramps; heat exhaustion; heat stroke	No specific threshold set**

*Fever defined as a temperature of 38 °C/100.4 °F or higher.

**The point at which to respond is based on discussions among the team
- Reporting forms: Developed for data capture at sentinel sites. Forms were case-based and included: name, age, sex, country of origin, province of origin, zone location in Honiara, syndrome, malaria smear result and whether laboratory sample(s) had been taken and sent to the laboratory.
- Web-based database: Developed for data entry, storage and analysis. This was hosted on an SPC server in Noumea.
- Data flow: Moved from aggregated reporting on a weekly basis to case-based reporting on a daily basis. Each afternoon sentinel sites completed the daily reporting form that was collected by the surveillance team the following morning and entered into the web-based system. Data were exported by SPC staff for analysis and preparation of daily situational reports that were returned to the Honiara team for vetting and dissemination.
- Response: For most syndromes the response point was based on team discussion. Exceptions were WD, F&J and AFN where a single case was investigated immediately (**Table 1**).
- Training: Nurses from sentinel sites were trained on the ESS system case definitions and reporting; the response team was trained on outbreak investigation.
- Feedback: Regular feedback of surveillance results was provided to sentinel sites to explain how data were used to prevent and respond to potential outbreaks.

### Stage 3 – sustainability

Towards the end of the ESS period, a focus group and a series of key informant interviews were conducted with staff who were involved in data collection, entry and analysis. The purpose of these sessions was to discuss the strengths and challenges of the system, lessons learnt about it and to explore how elements of the system could be sustained.

## Results

### Epidemiological findings

For the period of ESS, 1668 patients presented with one or more syndromes across the 15 sentinel sites. The average daily number of cases seen with one or more syndromes was 60; this decreased from 67 in the first half of the period to 52 in the last half of the period. Of those with one or more syndromes, 804 (48%) were female and 864 (52%) were male. The mean age of these cases was 13.2 years (range: 1 month to 82 years); 229 (13.7%) cases were infants aged less than 1 year and 803 (48%) were children aged less than 5 years.

The results are described by syndrome in **Table 2**. Of the patients with one or more syndromes, ILI was the most common syndrome (*n* = 727, 44%), followed by PF (*n* = 402, 24%), NWD (*n* = 387, 23%) and AFR (*n* = 204, 12%). Cases for each of the four most frequently occurring syndromes were relatively equally distributed between the sexes and had a broad age range with a mean age of between 12 and 15 years.

**Table 2:** Summary of enhanced syndromic surveillance cases, Solomon Islands, 25 June to 22 July 2012

Syndrome	Number of cases	Average number of cases per day	Percentage female	Mean age (range)	Percentage < 1 year	Percentage < 5 years
Influenza-like illness (ILI)	727	26	46% (*n* = 337)	13.7 (0.1–76)	16% (*n* = 114)	49% (*n* = 358)
Prolonged fever (PF)	402	14	50% (*n* = 199)	12.1 (0.1–64)	13% (*n* = 54)	49% (*n* = 198)
Non-watery diarrhoea (NWD)	387	14	49% (*n* = 188)	12.4 (0.1–82)	16% (*n* = 60)	61% (*n* = 235)
Acute fever and rash (AFR)	204	7	54% (*n* = 111)	12.9 (0.2–60)	6% (*n* = 13)	22% (*n* = 44)
Watery diarrhoea (WD)	91	3	42% (*n* = 38)	14.9 (0.1–68)	12% (*n* = 11)	42% (*n* = 38)
Acute fever and neurological symptoms (AFN)	3	0.1	67% (*n* = 2)	2.1 (0.6–4)	33% (*n* = 1)	100% (*n* = 3)
Fever and jaundice (F&J)	3	0.1	33% (*n* = 1)	37.3 (22–50)	0% (*n* = 0)	0% (*n* = 0)
Heat-related illness (HRI)	1	0.04	100% (*n* = 1)	21	0% (*n* = 0)	0% (*n* = 0)

**Fig. 1:**
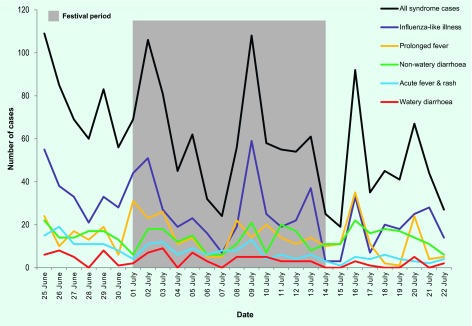
Number of syndrome cases seen over the ESS period, 25 June to 22 July 2012, Solomon Islands

The total daily number of syndrome cases peaked eight times throughout the surveillance period (**Fig. 1**); four peaks occurred during the FOPA. This peak pattern was largely due to an influx of patients on Mondays to clinics that had been closed for the weekend. The peaks were in ILI, PF and NWD that peaked at over 30, 20 and 15 daily cases, respectively, several times. AFR peaked at over 10 daily cases several times in the first half of the period. This was most likely due to the end of a rubella outbreak that had commenced before the ESS period.

Only a small number of the WD cases had laboratory samples taken. *Vibrio cholerae* was not found. Samples from NWD cases showed multiple enteric etiologies, including shigellosis and amoebiasis. Six cases of rubella and one case of dengue fever were confirmed during the period. A dengue fever preparedness and control plan for Solomon Islands was initiated. No other public health events of significance occurred.

### Focus group discussion and interviews

The focus group discussion and key informant interviews with staff who were involved in data collection, entry and analysis revealed several ESS areas that worked well and areas that were more challenging. Participants generally felt that the ESS was relatively simple and operated successfully and that it played an important role in the early detection of possible outbreaks.

Several strengths in the ESS were identified. Daily reporting from sites was generally carried out on time as was the data analysis and the preparation of daily situation reports. Staff felt that the regular feedback visits to the clinics were extremely useful; they provided an overview of the analysis results and checked the nurses’ understanding of the case definitions for quality assurance. The focus group discussion and informant interviews revealed that clinic staff attitudes changed markedly after the regular feedback visits started.

A major challenge noted in the ESS process was that some clinical staff were not clear on when and how to collect specimens. Many participants felt that some of the ESS improvements for early detection of and response to potential public health threats may not be sustainable beyond the mass gathering. Reasons given were limited human resources and limited transportation for samples, feedback visits and collecting data. Participants also said that updating Solomon Islands’ communicable disease policy and developing standard operational procedures for the SS system would be important for the sustainability of effective surveillance in Solomon Islands.

## Discussion

Through the analysis of the ESS that was operated at the FOPA, we have demonstrated that an ESS works relatively well for mass gatherings in resource-constrained settings. More than 1600 cases were captured across the 15 sentinel sites. The frequency of syndrome cases was tracked on a daily basis, triggering several outbreak investigations and informing public health promotion strategies.

### Strengths

The ESS provided the necessary elements for detecting and responding to disease outbreaks in a timely and effective manner. The existing SS system was expanded from one to 15 sentinel sites, from four to eight syndromes, from aggregated to case-based reporting and from weekly to daily reporting. A web-based database was established to expedite data entry, analysis and reporting. This enhanced information led to more efficient field investigations and responses. Therefore, it is possible that ESS contributed to early detection of diseases in Solomon Islands and in the broader region. While ESS for a mass gathering is resource-intensive, the improvements are not likely to be costly to sustain if electronic disease surveillance software is used. (*6*)

### Challenges and lessons learnt

There were several potential biases of the surveillance system. Graph peaks were largely influenced by an influx of patients on Mondays after clinics had been closed for the weekends. Some clinicians may have been more actively engaged in the system and thus more likely to report. There are likely to have been some misclassification of cases, particularly for those syndromes with similar case definitions.

The laboratory surveillance element of ESS was considered one of the main challenges. Several staff were unclear about specimen collection. Laboratory staff often did not communicate laboratory results back to sentinel sites, restricting the ability of clinic staff to undertake outbreak response. Laboratory and clinic staff should be given more training, including reinforcing the roles of each.

Many country delegate groups brought their own health personnel who often were the first people consulted by the delegation if they became ill. Consequently, clinics were not always accessed; thus, the ESS system may have missed a significant number of cases. A critical element in ensuring the sustainability of an ESS system is to have adequate lead in time for engagement and preparation (ideally at least 12 months). This should ensure that the appropriate policy and institutional frameworks, such as policy and standard operating procedures, are firmly in place in advance of the event. It will also enable the system users to become familiar with the system before the event.

## Conclusions

The 11th FOPA saw large crowds of people gather in Honiara for a public event. This implied an increased risk for the transmission of communicable disease, both at the event and across the region. An ESS system was used to strengthen the early detection and response to potential public health threats. The ESS system was considered a success, and it played an important role in the early detection of possible outbreaks. No major events of public health significance were experienced. Several lessons were learnt for the delivery of ESS in mass gathering scenarios. These included the importance of using a structured approach such as the one identified above, and engaging in planning for the SS of the event at least 12 months before ensure that appropriate and necessary policy and institutional frameworks are in place well before the event.
